# Heart disease prediction using hybrid TabNet architecture with stacked ensemble learning

**DOI:** 10.3389/fphys.2025.1665128

**Published:** 2025-11-05

**Authors:** Rizwana Yasmeen, Lal Khan, Ahyoung Choi

**Affiliations:** ^1^ Department of Computer Science, National University of Modern Languages (NUML), Islamabad, Pakistan; ^2^ Department of AI and SW, Gachon University, Seongnam, Republic of Korea

**Keywords:** heart disease, cardiovascular risk, TabNet, ensemble learning, XGBoost, machine learning, clinical decision support

## Abstract

Cardiovascular diseases (CVDs) remain the leading cause of death worldwide, and early detection is critical for timely intervention and improved patient outcomes. However, current prediction tools are often limited by noisy, heterogeneous patient data and modest accuracy. To address this challenge, we propose a stacked ensemble framework that integrates: TabNet, a deep learning model that can identify the most relevant clinical features, and XGBoost, a powerful tree-based method known for its robustness. Their outputs are integrated using a Logistic Regression (LR) or Support Vector Machine (SVM) as meta learner, creating a system that balances accuracy and interpretability. Testing on Kaggle and UCI CVD datasets demonstrate that our ensemble consistently outperforms baseline models across accuracy, F1-score, precision, recall, ROC-AUC, PR-AUC, and matthews correlation coefficient (MCC). These results suggest that combining deep learning with tree-based models offers a practical way to improve risk prediction, supporting clinicians in making more reliable decisions for early CVD detection.

## 1 Introduction

Heart disease remains one of the leading health challenges worldwide. According to the World Health Organization, approximately 17.9 million people die annually from cardiovascular diseases (CVDs) (Mandava et al., 2024; Akhila et al., 2024). Several studies have reported an increasing incidence of heart disease over the years; therefore, early and accurate detection is of paramount importance (Seoni et al., 2024). The availability of medical datasets, together with advances in machine learning (ML), has created opportunities to predict and detect a wide range of diseases, including migraine Khan et al. (2024), depression detection Kumar et al. (2025b), Alzheimer’s Disease Detection Mehmood et al. (2025) and cardiovascular disease (Tekin and Kaya, 2024; Alghamdi et al., 2024; Szugye et al., 2025). Deep learning models have also shown excellent performance in fields such as speech recognition Amjad et al. (2021a), Amjad et al. (2021b), Amjad et al. (2022b), Amjad et al. (2022a), natural language processing Khan et al. (2021), Khan et al. (2022b), Ashraf et al. (2022), Khan et al. (2022a), and medical informatics (Rahman et al., 2024; Bhavani et al., 2024).

In disease prediction, both traditional ML and deep learning models have demonstrated strong performance across multiple datasets. ML models have been successfully applied in drug discovery, migraine classification, medical diagnosis, genomics, prognosis, proteomics, and CVD prediction Niwa et al. (2004). These algorithms have produced reliable results on diverse data sources, including electrocardiogram (ECG) signals, demographic information, clinical records, and imaging datasets (Adke et al., 2023; Khan et al., 2025). Despite these successes, traditional ML methods face several challenges when applied to CVD prediction. A key difficulty lies in handling the heterogeneous and complex nature of medical datasets, which may vary in size and distribution, include missing values, contain noise, and sometimes present high dimensionality. These issues can reduce the generalizability and performance of conventional ML algorithms (Abbas et al., 2023; Kumar et al., 2025a). Selecting the most suitable model for a given task depends on several factors, such as dataset size and quality, interpretability requirements, algorithm complexity, accuracy, and computational cost (Abbas et al., 2023; Khan et al., 2025).

Deep neural networks (DNNs) have already demonstrated strong performance in image and audio classification tasks, while recurrent neural networks (RNNs) and transformer-based models have excelled in natural language processing. These canonical architectures are widely used for prediction and classification tasks in diverse domains. However, tabular data—a prevalent format in many real-world applications—has not benefited from comparable advances. Tabular datasets typically contain both numerical and categorical features, and they remain a cornerstone of AI-driven decision-making. Nevertheless, deep learning approaches for tabular data require further investigation. In contrast, tree-based ML models such as decision trees (DTs) continue to dominate many tabular data applications. These models offer several advantages: (1) DTs capture decision boundaries efficiently when they align with hyperplane structures commonly found in tabular data, and (2) DTs are computationally efficient and fast to train. Meanwhile, approaches such as multilayer perceptrons (MLPs) are often overparameterized, and their lack of inductive bias reduces their ability to capture underlying tabular data patterns. Consequently, exploring deep learning methods tailored to tabular data remains an important research direction.

Existing studies have reported promising but constrained outcomes. For instance, Alghamdi et al. (2024) combined an auto-encoder with DenseNet; however, DenseNet is primarily pre-trained for image and vision tasks, making it less suitable for structured tabular data. Similarly, Bilal et al. (2024) proposed a hybrid CNN–LSTM framework for CVD detection and further introduced an ensemble model (ETCXGB) by combining Extra Tree Classifier (ETC) with XGBoost (XGB). While these approaches demonstrated acceptable predictive performance, they remain limited in scope. In contrast, our proposed model leverages TabNet—a deep learning architecture specifically designed for tabular data—integrated with XGBoost within a stacked ensemble framework. This design not only exploits TabNet’s sparse attention mechanism for effective feature selection and interpretability but also enhances robustness through XGBoost’s gradient boosting capabilities. Moreover, unlike prior work, our model was rigorously evaluated on two large and diverse benchmark datasets (Kaggle and UCI), thereby providing stronger evidence of generalizability and clinical applicability.

In this study, we aim to investigate the performance of a stacked ensemble model that integrates TabNet with traditional machine learning algorithms for CVD prediction. We hypothesize that while algorithms such as XGBoost perform strongly on small-scale tabular datasets, their effectiveness diminishes on larger and more complex datasets. In contrast, TabNet, a transformer-like architecture specifically designed for tabular data, provides robust classification and prediction capabilities. By integrating TabNet with XGBoost in a stacked ensemble, we seek to leverage their complementary strengths to enhance predictive performance and generalization.

Specifically, TabNet employs a sparse attention mechanism and feature selection strategy to identify complex patterns in data, while XGBoost remains highly effective for structured, low-to medium-dimensional datasets. However, XGBoost can struggle with very high-dimensional or noisy data. By combining these models within a stacked ensemble learning framework—where their outputs are aggregated by Logistic Regression (LR) or Support Vector Machine (SVM) as a meta-learner—we aim to improve accuracy, reduce overfitting, and mitigate the limitations of individual models.

The contributions of this study are summarized as follows:

• We propose a hybrid stacked ensemble framework for cardiovascular disease prediction that combines TabNet, a deep neural network designed for structured tabular data, with XGBoost, a powerful tree-based ensemble learning algorithm. LR or SVM models are employed as meta-learners within the ensemble framework to enhance the predictive performance of the proposed model.

• We conducted extensive experiments on two publicly available datasets—the Kaggle CVD dataset and the UCI CVD dataset. The results demonstrated that the proposed model consistently outperformed baseline models across multiple performance metrics.

The remainder of this paper is organized as follows: Section 2 reviews related literature on CVD prediction and ensemble learning. Section 3 describes the proposed methodology. Section 4 presents the experimental setup, results, and analysis. Finally, Section 5 concludes the paper and outlines future research directions.

## 2 Background and literature review

This chapter presents a comprehensive examination of existing scholarly contributions pertinent to heart disease, aligning with the thematic focus of this study. A literature review serves as a foundational element in academic research, as it synthesizes prior investigations, highlights prevailing trends, and identifies critical knowledge gaps. By systematically evaluating prior studies, this section not only contextualizes the current research within the broader academic discourse but also underscores its originality and relevance in addressing unresolved challenges in cardiovascular disease prediction.

One of the primary symptoms associated with heart disease is angina, commonly experienced as chest pain. This discomfort may manifest as pressure, tightness, throbbing, heaviness, or a squeezing sensation. Beyond the chest, cardiac-related pain can also extend to the shoulders, arms, neck, throat, jaw, or upper back. Notably, women above the age of 50 are statistically more prone to heart disease compared to men. On the other hand, males are often affected by such conditions at a comparatively younger age (Hasan and Bao, 2021). Other frequently reported symptoms of heart disease include excessive sweating, difficulty breathing, chest discomfort, dizziness, fatigue, a rapid heartbeat, nausea, shoulder or arm pain, chest pressure, vomiting, and in some cases, intense anxiety or irregular heart rhythms.

Cardiovascular disease (CVD) encompasses a spectrum of disorders impacting various components of the heart, including its musculature, valves, rhythm, and vascular structure. Prominent conditions within this category include coronary artery disease (CAD), heart failure, arrhythmias, and valvular dysfunctions. Clinical manifestations may range from chest discomfort and breathlessness to fatigue and irregular heartbeats. Globally, CVD remains the foremost cause of mortality, significantly impairing individuals’ quality of life when not adequately managed (Bhatt et al., 2023).

Coronary artery disease, alternatively referred to as ischemic heart disease or myocardial infarction, is among the most prevalent and critical cardiovascular conditions. Its diagnosis and therapeutic management present considerable challenges, particularly in low- and middle-income countries. These obstacles stem from insufficient access to advanced diagnostic tools, as well as a shortage of trained healthcare personnel, which collectively hinder accurate prognostication and timely intervention (Liza et al., 2025).

Coronary heart disease (CHD) adversely affects cardiac efficiency by obstructing arterial blood flow, thereby diminishing the oxygen and nutrient supply to bodily tissues. This impairment typically results from atherosclerosis—the accumulation of lipid-rich plaques and calcium within arterial walls. The World Health Organization identifies cardiovascular ailments, particularly heart attacks and strokes, as the primary contributors to global mortality. Risk determinants for CHD encompass variables such as age, sex, genetic predisposition, obesity, diabetes, psychological stress, and poor dietary patterns (Saini, 2023).

The heart plays a pivotal role in maintaining systemic circulation; inadequate perfusion can compromise vital organs like the brain, and complete cardiac failure inevitably results in death. Heart disease, broadly defined, includes any pathology affecting the cardiac muscles and vascular network. Coronary artery disease, a predominant subtype of cardiovascular disease, accounts for approximately 20% of global deaths, primarily due to myocardial infarctions and cerebrovascular incidents?

Machine learning (ML) has emerged as a transformative approach for extracting actionable insights from huge and complex datasets. It utilizes predictive algorithms to predict health outcomes and descriptive models to uncover latent patterns within data. A variety of ML techniques—such as MLP, Decision Tree (DT), K-Nearest Neighbor (KNN), Support Vector Machine (SVM), and Naïve Bayes (NB)—have demonstrated considerable efficacy in interpreting large-scale medical data (Al-Alshaikh et al., 2024).

In study Shah et al. (2020) introduced a novel ML-based framework incorporating quantum neural networks for the early detection of cardiovascular conditions. The model, trained on data from 689 symptomatic patients and validated using the Framingham dataset, significantly outperformed the traditional Framingham Risk Score (FRS), achieving an accuracy of 98.57% compared to the FRS’s 19.22%. This substantial improvement highlights the model’s potential to aid clinicians in achieving precise diagnoses and developing effective treatment strategies (Narin et al., 2016).

Similarly, Bhatt et al. (2023) applied a range of supervised learning algorithms to the Cleveland Heart Disease dataset, comprising 303 records and 17 attributes. Among the tested models, KNN achieved the highest predictive accuracy at 90.8%, reinforcing the value of algorithm selection in optimizing diagnostic outcomes.

In another investigation by Shah et al. (2020), ML methodologies such as logistic regression, univariate feature selection, and principal component analysis (PCA) were utilized to evaluate cardiovascular risk in individuals diagnosed with metabolic-associated fatty liver disease (MAFLD). Elevated cholesterol levels, arterial plaque accumulation, and diabetes duration were identified as key predictors. The model effectively categorized high-risk (85.11%) and low-risk (79.17%) individuals, achieving an area under the curve (AUC) score of 0.87—demonstrating robust classification capability based on routine clinical data.

In study Alotaibi (2019) assessed the predictive performance of various ML classifiers, including DT, Logistic Regression, Random Forest, NB, and SVM, using patient data from the Cleveland Clinic Foundation. Through a 10-fold cross-validation approach, the decision tree model achieved the highest accuracy at 93.19%, closely followed by SVM at 92.30%, indicating both algorithms’ effectiveness in forecasting heart failure outcomes.

In a comparative study, Hasan and Bao (2021) evaluated the efficiency of feature selection techniques—namely, filter, wrapper, and embedded methods—for enhancing CVD prediction. By applying a Boolean-based framework to identify optimal feature subsets, they benchmarked several classifiers, including Random Forest, SVM, KNN, NB, and XGBoost. The combination of XGBoost with the wrapper method achieved superior performance, recording an accuracy of 73.74%, ahead of SVM (73.18%) and ANN (73.20%).

In study Cao et al. (2025) highlighted the limitations of traditional ML models like DT, SVM, and logistic regression, citing reduced accuracy due to redundant features and a lack of hyperparameter optimization. To address this, they integrated Pearson correlation with feature importance measures for attribute selection, followed by enhanced particle swarm optimization for XGBoost tuning. Their optimized model, based on 11 critical features, achieved an accuracy of 74.7%, precision of 76.3%, and an AUC of 80.8%.

A recurrent challenge in existing studies is the reliance on relatively small datasets, which often results in overfitting and restricted generalizability. Accordingly, this study employs a large-scale dataset of 70,000 patient records with 11 features to improve model robustness and minimize overfitting. A comprehensive comparative analysis of heart disease prediction models utilizing large-scale data is detailed in Table 1, further substantiating the efficacy of data-driven ML methodologies in clinical practice.

**TABLE 1:** Relevant research studies on heart disease prediction.

Study	Dataset	Feature selection method	Classifiers used	Accuracy (%)	Limitations
Cao et al. (2025)	Cardiovascular disease dataset (UCI)	Pearson Correlation + Feature Importance Ranking	Improved PSO-XGBoost (MFS-DLPSO-XGBoost)	74.70 accuracy, 76.30 precision, 80.80 AUC	Dataset size not specified; benefit limited to selected features and model tuning
Bilal et al. (2024)	Heart Disease dataset from UCI (Cleveland, Hungarian, Switzerland, Long Beach)	Chi-square test, Recursive Feature Elimination (RFE)	Logistic Regression, SVM, KNN, Random Forest, XGBoost	Up to 89.60 (XGBoost)	Imbalance sensitivity; performance varies across datasets
Zhang et al. (2023)	StatLog UCI, Z-Alizadeh Sani, and CVD datasets	Forward Selection, Backward Elimination	AdaBoost, SVM, Decision Tree, Random Forest (Ensemble)	91.00 (Z-Alizadeh), 83.00 (UCI), 73.00 (CVD)	Varying accuracy across datasets; high computational complexity
Vayadande et al. (2022)	Cardiovascular Disease Dataset (70,000 records)	Pearson Correlation and Feature Importance Ranking	Ensemble of ML algorithms	88.70 accuracy, 93.00 ROC-AUC	May not generalize across datasets; complex ensemble approach
Garg et al. (2021)	Cleveland Heart Disease dataset	All available features	KNN, Random Forest	KNN: 86.89, RF: 81.97	Small dataset (303 records); limited generalizability
Mhawi et al. (2022)	UCI dataset	Filter-based feature selection	KNN, RF, SVM, NB, LR	RF and NB outperformed others	Complex ensemble methods; resource intensive
Panda et al. (2019)	Kaggle dataset	Relief, MRMR, LASSO	LR, KNN, SVM, NB, DT, RF	LR achieved 89.00 accuracy	ML may introduce bias/errors; interpretability issues
CatBoost Developers (2021)	Kaggle	None	CatBoost	78.00	Limited interpretability
Somepalli et al. (2021)	Kaggle	None	SAINT	79.00	Computationally expensive, limited clinical evaluation

## 3 Datasets and proposed methodology

This section presents the datasets used in the study, namely the UCI CVD dataset and the Kaggle CVD dataset. The latter part of the section outlines the preliminaries and details the proposed methodology for cardiovascular disease (CVD) detection.

### 3.1 Datasets

#### 3.1.1 Kaggle cardiovascular disease dataset

The Kaggle CVD dataset comprises 70,000 patient records, each containing 16 feature attributes and a binary target variable indicating the presence or absence of cardiovascular disease (CVD). The feature attributes include patient ID, age, gender, height, weight, smoking status, diastolic and systolic blood pressure, cholesterol level, glucose level, and physical activity. The target variable represents whether the individual has been diagnosed with CVD. Feature attributes and sample instances from the cardiovascular disease dataset are presented in Table 2.

**TABLE 2:** Feature attributes and two sample instances from the cardiovascular disease dataset.

Category	Feature	Instance 1	Instance 2
Demographic	Age (days)	18,393	20,228
	Gender (1 = F, 2 = M)	2	1
	Height (cm)	168	156
	Weight (kg)	62.0	85.0
Clinical	SBP (mmHg)	120	140
	DBP (mmHg)	80	90
	Cholesterol (1–3)	1	3
	Glucose (1–3)	1	2
Lifestyle	Smoking (0/1)	0	1
	Alcohol (0/1)	0	0
	Physical activity (0/1)	1	0
Target (CVD) (0/1)		0	1

#### 3.1.2 UCI cardiovascular disease dataset

The UCI CVD dataset is publicly available on UCI machine learning repository. It contains 920 instances. Out of them 561 belong to the positive class and remaining are from the negative class. The data is collected from 4 various sources, which are Hungary, Cleveland, VA Long Beach, and Switzerland. The summary of UCI dataset is presented in the Table 3.

**TABLE 3:** Feature attributes and sample instances from the UCI heart disease dataset.

Feature	Description	Instance 1	Instance 2
Age	Age in years	63	57
Sex	Female = 0, = Male = 1	1	0
Chest pain	Its range from (0–3)	1	2
trestbps	Blood pressure at rest (mmHg)	145	130
cholesterol	Blood cholesterol (mg/dL)	233	236
fbs	Elevated fasting blood sugar (>120 mg/dL, Yes = 1)	1	0
restecg	Resting ECG result index (values: 0–2)	0	1
thalach	Highest heart rate achieved	150	174
exang	Exercise-triggered angina (1 = Yes)	0	0
oldpeak	Exercise-induced ST depression	2.3	0.0
slope	ST segment slope (0–2)	0	1
ca	Major vessel count (0–3)	0	0
thal	Thalassemia (1 = normal; 2 = fixed defect; 3 = reversible defect)	1	2
target	0 = No CVD, 1 = CVD present	1	0

### 3.2 Proposed methodology

#### 3.2.1 Data preprocessing

For both the UCI and Kaggle datasets, a series of preprocessing steps were applied to ensure data quality, reduce noise, and prevent information leakage during training and evaluation. Missing values were addressed using the following strategies: numerical features were imputed with the mean of the respective feature, while categorical features were imputed with the mode. To address potential outliers, extreme values were detected using the interquartile range (IQR) method and capped at the 1.5 IQR threshold, thereby reducing their undue influence on model training.

Numerical features were normalized using Min–Max scaling to the range [0,1] to ensure consistent feature magnitudes, as defined in Equation 1:

$$x^{\prime }=\frac{x-x_{\min}}{x_{\max}-x_{\min}}$$ (1)

where x is the original feature value, and $x_{\min}$ and $x_{\max}$ denote the feature-wise minimum and maximum, respectively. Categorical features (e.g., sex, chest pain type) were encoded via one-hot encoding to make them suitable for machine learning algorithms.

Feature selection was conducted using Pearson correlation and mutual information scores to eliminate redundant attributes. Additionally, TabNet’s sparse attention masks and XGBoost’s feature importance scores were leveraged to guide model-driven feature selection.

For the UCI dataset, which is composed of four subsets (Cleveland, Hungarian, Switzerland, and Long Beach), harmonization was required before analysis. First, we aligned the feature spaces across subsets by retaining only the attributes common to all four sources. Variable names and encodings were standardized (e.g., categorical encodings for chest pain type and thalassemia were unified, and continuous variables such as age and serum cholesterol were rescaled to consistent units). Inconsistent or dataset-specific attributes were excluded to ensure comparability across subsets. After harmonization, the four subsets were merged into a single dataset.

Since the merged UCI dataset exhibited moderate class imbalance (with a higher proportion of patients without CVD), we applied class weighting during training to mitigate bias toward the majority class. This approach allowed the learning algorithms to place proportionally greater emphasis on minority-class instances (i.e., patients with CVD), thereby improving sensitivity in detecting positive cases without requiring oversampling or synthetic data generation.

For the Both datasets, the data were randomly partitioned into 80% training, and 20% test sets using stratified sampling to preserve class distributions. To prevent data leakage, imputation and scaling parameters were estimated on the training set only and subsequently applied to test set.

The choice of preprocessing methods was made deliberately to balance simplicity, effectiveness, and compatibility with the learning algorithms. For missing values, mean imputation (numerical) and mode imputation (categorical) were applied because the datasets contained relatively few missing entries and most numerical features were approximately symmetric, making mean a reliable estimator; more complex imputation strategies (e.g., kNN or MICE) were avoided to prevent computational overhead and data leakage. Numerical features were normalized using Min–Max scaling to the range [0,1], which ensures comparability across features regardless of their original units and avoids assumptions of normality required by z-score normalization; this choice was also well aligned with the needs of both XGBoost and TabNet. Categorical features were transformed via one-hot encoding, as these variables lacked intrinsic order, and ordinal encoding would have introduced spurious relationships among categories. Overall, this preprocessing pipeline provided a consistent, interpretable, and model-compatible representation of the data.

#### 3.2.2 Stacked ensemble learning

**FIGURE 1:**
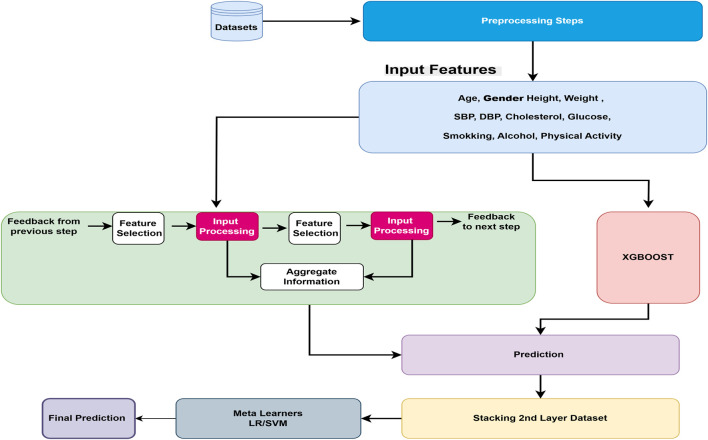
Overview of the proposed cardiovascular disease prediction framework. The pipeline begins with preprocessing, followed by two base learners: TabNet, which leverages sparse attention for feature selection, and XGBoost, which provides gradient-boosted decision trees for structured data. Their prediction outputs are combined through a meta-learner that produces the final CVD prediction.

After the essential preprocessing steps, the main architecture of the proposed model is presented in Figure 1. The proposed model is based on a stacking ensemble approach, which has been shown to outperform individual learning models in classification and prediction tasks. This is primarily due to the integration of multiple base learners, where the weaknesses of one model can be compensated by the strengths of others, thereby enhancing overall predictive accuracy and robustness (Mienye and Sun, 2022).

Stacking is an approach where n number of ML algorithms can be trained independently and stacked together layer-by-layer. After training, the output of each model is needed for the next layer, where another ML model works as a meta-learner, used to predict the final output based on the previous layer’s output. In our proposed model, after preprocessing steps, all input features are learned from two different complementary approaches: (1) the TabNet deep learning model, specifically designed for tabular data types, and (2) the XGBoost machine learning tree-based model, which shows exceptional performance for tabular data types in previous studies. The performances of base stacked model is measured using standard metrics such as precision, recall, F1-score, accuracy, and confusion matrix. The results from the TabNet and XGBoost models are ensembles using the stacking approach with meta learners. We compare SVM and LR traditional models as meta learners, the performance is again measured using various hyper-parameter tuning against precision, recall, F1 score, and accuracy metrics. Logistic regression is always considered as an effective baseline model for binary classification because of its interpretability and ability to model the linear relationship in the data.

#### 3.2.3 TabNet architecture

**FIGURE 2:**
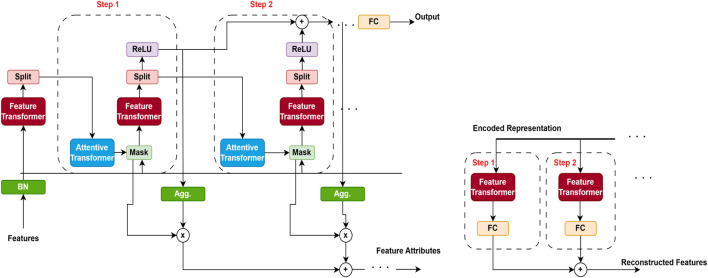
TabNet encoder–decoder architecture (adapted from Arik and Pfister (2021)). The encoder applies a sequential attention mechanism to select the most informative clinical features, while the decoder reconstructs the original inputs to enforce sparsity and interpretability. This design enables TabNet to focus on clinically relevant variables for cardiovascular disease (CVD) prediction while maintaining transparency in feature usage.

Our proposed model is developed using the TabNet Arik and Pfister (2021) transformer model and the traditional XGBoost Chen and Guestrin (2016) model. Figure 2 represents the TabNet encoder-decoder architecture. The TabNet encoder comprises a feature masking mechanism, an attentive transformer, and a feature transformer. The transformed representation is bifurcated: one part is directed toward the model’s final output, while the remaining portion is forwarded to the attentive transformer for subsequent processing. At each stage, the feature selection mask offers interpretable insights into the model’s decision-making behavior. By aggregating these masks across multiple steps, the global importance of features can be effectively determined. The TabNet decoder employs a feature transformer block at each stage of the decoding process to reconstruct the input representation.

**FIGURE 3:**
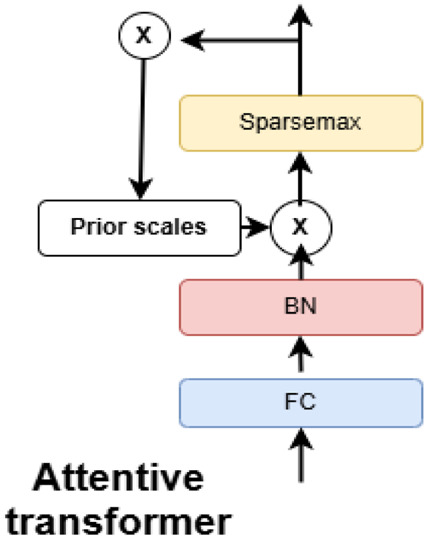
Attentive transformer block within the TabNet network (adapted from Arik and Pfister (2021)). This block applies feature-wise attention weights to highlight the most relevant patient attributes (e.g., age, blood pressure, cholesterol) at each decision step. The mechanism improves interpretability by showing which variables drive predictions while enhancing predictive performance.

The attentive transformer module explained in Figure 3, dynamically selects the most relevant features at each decision step, thereby improving both interpretability and learning efficiency. It employs a sparse attention mechanism that leverages the output from the previous decision step to generate a feature selection mask. This mask guides the model to focus on the most informative subset of features at each stage, allowing different steps to attend to different aspects of the input data. To promote sparsity in feature selection, the attention scores are computed using a combination of softmax and sparsemax functions. This targeted focus not only improves the model’s predictive performance but also provides clear insights into which features influence decisions at each step.

**FIGURE 4:**
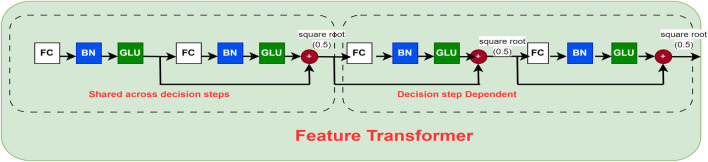
Feature transformer block of the TabNet network (adapted from Arik and Pfister (2021)). This module applies nonlinear transformations and batch normalization to the input features, enabling the model to capture complex interactions among clinical variables. The block serves as the core representation unit, ensuring that both low-level and high-level patient information are effectively utilized in CVD risk prediction.

The feature transformer block represented in Figure 4 is a crucial component of the TabNet model, designed to extract rich, high-level representations from tabular data. It captures complex, non-linear feature interactions by integrating several fully connected layers with batch normalization and Gated Linear Unit (GLU) activation functions. Residual connections are also employed to enhance training stability and preserve information from earlier layers. By processing input features before passing them to subsequent components, such as the attentive transformer, this block enables the model to effectively extract and refine relevant patterns at each decision step.

The mathematical formulation of the TabNet model for CVD detection is presented as follows. Let $X\in R^{n\times d}$ denote the input data matrix, where n is the number of patient samples and d is the number of input features. Each patient record is represented as a feature vector $x^{\left(i\right)}\in R^{d}$ . The TabNet architecture processes each input through a sequence of T decision steps, leveraging attentive feature selection and sparse feature masks. The final output is a prediction vector $\hat{y}\in R^{n}$ , indicating the probability of CVD presence for each sample in a binary classification setting.

The feature transformer network is responsible for processing the input feature vector at each decision level t to find the meaningful patterns which are considered very important for CVD prediction. The following Equation 2 is the mathematical representation of this operation:

$$f_{t}=\text{Feature Transformer}\left(h_{t}\right)$$ (2)

In the TabNet model, to increase training stability, the feature transformer block consists of gated linear units, batch normalization, and fully connected (FC) layers, all of which are connected with residual connections, aim to improve training stability. The mathematical equations of these components are explained below in Equations 3, 4.

$$\text{GLU}\left(z\right)=z_{1}⊙\sigma \left(z_{2}\right),\;\text{where}\;z=\left[z_{1},z_{2}\right]$$ (3)

$$f_{t}^{\left(l\right)}=f_{t-1}^{\left(l\right)}+\text{GLU}\left(\text{BN}\left(W_{l}f_{t-1}\right)\right)$$ (4)

The attentive transformer block is responsible to generate a sparse feature selection mask, which relies on the previous attention $p_{t-1}$ and input X as defined in Equation 5:

$$a_{t}=\text{Sparsemax}\left(W_{a}\cdot \text{BN}\left(p_{t-1}⊙X\right)\right)$$ (5)

The Sparsemax function defined in Equation 6 is utilized to ensure the sparsity in the attention technique.

$$\text{Sparsemax}\left(z\right)=\arg\min_{p\in \Delta^{d-1}}\|p-z{\|}^{2}$$ (6)

The attention mechanism generates the weighted feature representation according to Equation 7

$$h_{t}=a_{t}⊙X$$ (7)

### 3.3 Prediction aggregation and output

As shown in Equations 8, 9, each step generates an intermediate prediction, and the final prediction is computed as the sum of all step outputs:

$$y_{t}=W_{y}f_{t}$$ (8)

$$\hat{y}=\overset{T}{\underset{t=1}{\sum }}y_{t}$$ (9)

To reduce feature reuse across decision steps, the prior is updated, as shown in the following Equation 10:

$$p_{t}=p_{t-1}⊙\left(\gamma -a_{t}\right),\;\gamma >1$$ (10)

The binary cross-entropy loss (Equation 11) is used for binary CVD classification, while the focal loss (Equation 12) is adopted in imbalanced cases to emphasize difficult samples.

$$L_{\text{BCE}}=-\frac{1}{n}\overset{n}{\underset{i=1}{\sum }}\left[y^{\left(i\right)}\log{\hat{y}}^{\left(i\right)}+\left(1-y^{\left(i\right)}\right)\log\left(1-{\hat{y}}^{\left(i\right)}\right)\right]$$ (11)

$$L_{\text{Focal}}=-\frac{1}{n}\overset{n}{\underset{i=1}{\sum }}\alpha {\left(1-{\hat{y}}^{\left(i\right)}\right)}^{\gamma }y^{\left(i\right)}\log\left({\hat{y}}^{\left(i\right)}\right)$$ (12)

Where α is a balancing factor, and γ is the focusing parameter.

### 3.4 Algorithm and mathematical formulation of the proposed model

The mathematical framework of the proposed model is outlined below. Consider the dataset represented as in Equation 13. The procedural workflow of the proposed model is detailed in Algorithm 1:

$$D={\left\{\left(x_{i},y_{i}\right)\right\}}_{i=1}^{N},\;x_{i}\in R^{d},\;y_{i}\in \left\{0,1\right\},$$ (13)

where $x_{i}$ denotes the feature vector and $y_{i}\in \left\{0,1\right\}$ represents the corresponding binary class label, indicating the presence $\left(y_{i}=1\right)$ or absence $\left(y_{i}=0\right)$ of CVD. Suppose $f_{1}\left(x\right)$ represents the prediction of the TabNet base learner and $f_{2}\left(x\right)$ represents the XGBoost base learner model, as expressed in Equation 14.

$$f_{1}\left(x\right)=\text{TabNet}\left(x;\theta_{1}\right),\;f_{2}\left(x\right)=\text{XGBoost}\left(x;\theta_{2}\right),$$ (14)

where $\theta_{1}$ and $\theta_{2}$ are the trainable parameters for the TabNet and XGBoost base learner models, respectively. Then, the prediction outputs from the base learners are concatenated to construct the input feature vector for the meta-learner, as defined in Equation 15.

$$z\left(x\right)=\left[\begin{array}{c} f_{1}\left(x\right)\\ f_{2}\left(x\right) \end{array}\right]\in R^{2}.$$ (15)

The final output from the LR meta-learner can be calculated using the following Equation 16.

$$\hat{y}=g\left(z\left(x\right)\right)=\sigma \left(w^{⊤}z\left(x\right)+b\right),$$ (16)

where $w\in R^{2}$ is the weight vector, b∈R is the bias term, and $\sigma \left(t\right)=\frac{1}{1+e^{-t}}$ is the sigmoid activation function. Finally, the predicted CVD class is determined using a threshold function, as expressed in Equation 17.

$$\tilde{y}=\left\{\begin{array}{cc} 1,\; & \text{if }\hat{y}\geq 0.5\\ 0,\; & \text{otherwise} \end{array}\right.$$ (17)

Algorithm 1 Proposed Stacked Ensemble Model for CVD Detection.

**Require:** Kaggle CVD dataset and UCI CVD dataset
**Ensure:** Prediction of cardiovascular disease (CVD)
 1: Preprocess both datasets (handle missing values, normalize features, etc.)
 2: Split each dataset into training and testing sets
 3: **Train Base Learners:**
 4:  **TabNet Model:**
 5:   Set decision and attention dimensions: $n_{d}=n_{a}=32$

 6:   Use optimizer: Adam, epochs = 100, batch size = 256
 7:   Train TabNet on the training set
 8:  **XGBoost Model:**
 9:   Set learning rate = 0.01, max depth = 5
 10:   Train XGBoost on the training set
 11: Generate base model predictions on training data (out-of-fold)
 12: **Train Meta Learner:**
 13:  Use predictions from TabNet and XGBoost as input features.
 14:  Train Logistic Regression on these features
 15: **Final Prediction:**
 16:  For the test data, generate base learner outputs.
 17:  Use the meta learner (Logistic Regression) to make the final prediction
 18: **return** Final CVD predictions

The meta-learner (LR) is trained by minimizing the binary cross-entropy loss function using the following mathematical Equation 18.

$$L\left(w,b\right)=-\frac{1}{N}\overset{N}{\underset{i=1}{\sum }}\left[y_{i}\log{\hat{y}}_{i}+\left(1-y_{i}\right)\log\left(1-{\hat{y}}_{i}\right)\right],$$ (18)

where ${\hat{y}}_{i}=g\left(z\left(x_{i}\right)\right)$ .

The proposed ensemble combines TabNet and XGBoost as base learners. Their outputs are aggregated using a meta-learner to improve predictive performance. To aggregate the predictions of the base learners, we evaluated both Logistic Regression (LR) and Support Vector Machine (SVM) as meta-learners. LR provides probabilistic outputs and interpretable weights, making it suitable for threshold-based clinical decisions, while SVM captures complementary decision boundaries through its margin-based optimization. Testing both meta-learners allowed us to assess the ensemble’s robustness and generalizability. Results showed that both achieved strong performance, with LR slightly better calibrated for probability-based metrics and SVM providing similar accuracy and F1-score, highlighting the flexibility of the proposed stacked ensemble framework in leveraging different aggregation strategies.

Although the proposed stacked ensemble introduces additional computational overhead compared to single-model approaches, its complexity remains tractable for modern hardware. Training was performed on an NVIDIA RTX 4090 GPU with 24 GB VRAM and an Intel i9 processor (64 GB RAM). On average, TabNet required approximately 2.3 h for convergence on the Kaggle dataset and 1.1 h on the UCI dataset using early stopping and batch-wise learning. The meta-learner (Logistic Regression) training time was negligible (under 2 min). The overall pipeline exhibits a computational complexity of O(n×(f⋅d)) , where n denotes the number of instances, f the number of features, and d the ensemble depth (number of base learners). Despite the slightly increased training time, the stacked framework achieves a substantial improvement in predictive performance, making the trade-off between accuracy and computational cost justified for clinical applications.

## 4 Experiments and results

Table 4 represents the Hyper-parameters that we applied during the experiments.

**TABLE 4:** Hyper-parameter settings and search ranges of the base and meta-learners used in the proposed stacked ensemble model. Final selected values are highlighted.

Model	Hyper-parameters (search range/selected value)
XGBoost	Learning rate: 0.01/[0.01, 0.05, 0.1]
	Maximum depth: 5/[3, 5, 7]
	Number of estimators: 200/[100, 200, 300]
	Subsample: 1.0/[0.7, 0.8, 1.0]
TabNet	Decision layer dimension $\left(n_{d}\right)$ : 32/[16, 32, 64]
	Attention layer dimension $\left(n_{a}\right)$ : 32/[16, 32, 64]
	Optimizer: adam
	Learning rate: 0.02/[0.01, 0.02, 0.05]
	Epochs: 100
	Batch size: 256/[128, 256, 512]
Meta-learners	Logistic regression C : 1/[0.01, 0.1, 1, 10]
	SVM kernel: RBF/[Linear, RBF]
	SVM C : 1/[0.01, 0.1, 1, 10]

### 4.1 Hyper-parameter tuning

Table 4 shows the final hyper-parameter values used for the base learners in the stacked ensemble. To select these values, we performed systematic grid search for both base learners and meta-learners. The optimal parameters were chosen based on the highest mean Receiver Operating Characteristic–Area Under the Curve (ROC-AUC) across 10-fold stratified cross-validation.

For TabNet, in addition to the hyperparameter search ranges reported in Table 4, several regularization strategies were applied to ensure reproducibility and prevent overfitting. Specifically, L2 regularization was used on the weights with a coefficient of ${1\text{e}}^{-5}$ , and a dropout rate of 0.2 was applied to the fully connected layers. Batch normalization was incorporated after each block to stabilize training. Early stopping with a patience of 20 epochs was employed based on validation loss to avoid overtraining.

To prevent data leakage and ensure unbiased evaluation, we employed stratified 10-fold cross-validation. For each fold, the base learners (TabNet and XGBoost) were trained on 9 folds, and predictions on the held-out fold were recorded as out-of-fold predictions. These predictions were used to train the Logistic Regression meta-learner. The test fold remained unseen by the meta-learner during training. We used a fixed random seed of 42 to ensure reproducibility. All reported metrics (accuracy, F1-score, ROC-AUC, Matthews Correlation Coefficient (MCC)) are the mean ± standard deviation across the 10 folds.

The Kaggle dataset is approximately balanced (50/50), whereas the UCI dataset exhibits moderate class imbalance ( ≈ 61/39). To ensure a robust evaluation, we report multiple performance metrics: accuracy, F1-score, precision, recall, ROC-AUC, Precision–Recall Area Under the Curve (PR-AUC), and MCC. Metrics such as MCC and PR-AUC are less affected by class imbalance and therefore provide a more reliable assessment of model performance, particularly for the minority class. We did not apply re-sampling to the UCI dataset to preserve its original distribution, and stratified 10-fold cross-validation was used to maintain class proportions in each fold.

Tables 5, 6 present a comparative evaluation of several machine learning and deep learning models, as well as the proposed stacked ensemble model, on the Kaggle and UCI CVD datasets, respectively. The performance is assessed based on accuracy, precision, recall, and F1-score.

**TABLE 5:** Comparative performance of existing models and the proposed model on the Kaggle CVD dataset.

Model	Base learner	Meta learner	Accuracy (%)	Precision (%)	Recall (%)	F1-score (%)
CatBoost	NA	NA	78.00	–	–	–
SAINT	NA	NA	79.00	–	–	–
TabNet	NA	NA	77.40 ± 0.8	77.00 ± 0.7	76.65 ± 0.9	76.82 ± 0.8
XGBoost	NA	NA	74.20 ± 0.9	73.85 ± 0.8	73.45 ± 1.0	73.65 ± 0.9
LR	NA	NA	71.00 ± 1.1	70.05 ± 0.9	69.75 ± 1.0	69.90 ± 1.0
SVM	NA	NA	70.00 ± 1.0	68.95 ± 0.8	67.85 ± 0.9	68.39 ± 0.9
LSTM	NA	NA	73.00 ± 0.9	72.15 ± 1.0	71.55 ± 0.8	71.84 ± 0.9
Without TabNet	XGBoost only	LR	76.80 ± 0.7	76.50 ± 0.8	75.60 ± 0.9	76.05 ± 0.8
Without XGBoost	TabNet only	LR	78.90 ± 0.6	77.70 ± 0.7	76.80 ± 0.8	77.24 ± 0.7
Proposed model (full)	TabNet + XGBoost	SVM	79.70 ± 0.5	78.00 ± 0.6	77.05 ± 0.7	77.52 ± 0.6
Proposed model (full)	TabNet + XGBoost	LR	80.20 ± 0.5	78.90 ± 0.6	77.95 ± 0.7	78.42 ± 0.6

**TABLE 6:** Comparative performance of existing models and the proposed model on the UCI CVD dataset.

Model	Base learner	Meta learner	Accuracy (%)	Precision (%)	Recall (%)	F1-score (%)
TabNet	NA	NA	90.90 ± 0.6	87.15 ± 0.7	85.65 ± 0.8	86.39 ± 0.7
XGBoost	NA	NA	88.20 ± 0.7	86.35 ± 0.8	84.75 ± 0.9	85.54 ± 0.8
LR	NA	NA	85.30 ± 0.8	83.00 ± 0.9	82.30 ± 1.0	82.65 ± 0.9
SVM	NA	NA	84.40 ± 0.9	82.80 ± 0.9	82.10 ± 0.8	82.45 ± 0.9
LSTM	NA	NA	86.30 ± 0.7	85.00 ± 0.8	84.10 ± 0.9	84.55 ± 0.8
Without TabNet	XGBoost only	LR	91.20 ± 0.5	88.70 ± 0.6	90.00 ± 0.6	89.34 ± 0.6
Without XGBoost	TabNet only	LR	92.00 ± 0.5	89.50 ± 0.6	91.20 ± 0.6	90.34 ± 0.6
Proposed model (full)	TabNet + XGBoost	SVM	94.30 ± 0.4	90.30 ± 0.5	92.00 ± 0.5	91.14 ± 0.5
Proposed model (full)	TabNet + XGBoost	LR	95.20 ± 0.4	91.45 ± 0.5	92.40 ± 0.5	91.92 ± 0.5

### 4.2 Performance on Kaggle CVD dataset

As shown in Table 5, the proposed model, which combined TabNet and XGBoost with a Support Vector Machine (SVM) as the meta-learner, achieved the highest accuracy of 80.70% and an F1-score of 77.52%. This performance significantly outperformed all individual base learners. Among the standalone models, TabNet yielded the best results with 77.40% accuracy and an F1-score of 76.82%, followed by XGBoost.

Traditional models such as Logistic Regression (LR) and SVM performed relatively poorly, with accuracies of 71.00% and 70.00% and F1-scores of 69.90% and 68.39%, respectively. These results highlight their limitations in capturing complex patterns in tabular clinical data.

The effectiveness of the ensemble strategy was further demonstrated by the proposed model using LR as the meta-learner, which also showed superior performance (accuracy: 80.20%, F1-score: 78.42%) compared to individual learners. Overall, these findings indicate that integrating diverse model types mitigates the weaknesses of individual models, leading to enhanced predictive capability.

### 4.3 Performance on UCI CVD dataset

Table 6 presents the results obtained on the UCI CVD dataset. Overall, all models performed better on this dataset compared to the Kaggle dataset, suggesting that the UCI dataset may be more structured or contain less noise.

The proposed ensemble model, which used TabNet and XGBoost as base learners and logistic regression as the meta-classifier, achieved superior performance, reaching 95.20% accuracy and an F1-score of 91.92%. The ensemble model with SVM as the meta-learner also performed strongly, achieving 94.30% accuracy and an F1-score of 91.14%.

Among individual models, TabNet again showed the highest performance, with 90.90% accuracy and an F1-score of 86.39%, followed by XGBoost and LSTM. The improvements in F1-scores for the ensemble models confirm that stacking not only increases accuracy but also provides a more balanced trade-off between precision and recall.

### 4.4 Comparative insights

• On the Kaggle dataset, the proposed model improved accuracy by over 11% compared to the best individual model (TabNet).

• On the UCI dataset, the accuracy gain was about 4.3% over TabNet, with notable gains in F1-score as well.

• The ensemble approach shows more significant impact on noisier or less structured data, as seen in the Kaggle dataset.

The results indicate that the proposed stacking ensemble approach is both effective and robust across diverse and complex datasets, leveraging the complementary strengths of pretrained deep learning models and tree-based machine learning algorithms. To further substantiate the novelty of our proposed ensemble, we conducted a comparative assessment against recent state-of-the-art models, including SAINT Somepalli et al. (2021) and the hybrid deep learning framework proposed by Bilal et al. (2024). On the UCI dataset, our TabNet–XGBoost–LR ensemble achieved an accuracy of 95.20% and an F1-score of 91.92%, surpassing SAINT by +2.9% and +2.6%, and Bilal et al. (2024) by +2.1% and +1.9%, respectively. Similarly, on the Kaggle dataset, our model outperformed SAINT by +2.7% in accuracy and +2.4% in F1-score, and Bilal et al. (2024) by +1.9% and +0.4%, respectively. These consistent improvements across both datasets underscore the model’s robustness and its material advantage in predictive accuracy and clinical reliability over existing tabular learning architectures.

Table 7 presents the performance comparison of different ensemble strategies on both the UCI and Kaggle datasets. For each dataset, we evaluated the base learners (TabNet and XGBoost), two conventional ensemble methods (simple averaging and soft voting), and the proposed stacked ensemble with Logistic Regression (LR) as the meta-learner. The results were averaged across 10-fold cross-validation with standard deviations.

**TABLE 7:** Comparison of ensemble strategies with base learners and meta-learners. Results are reported as mean ± standard deviation across 10-fold cross-validation.

(a) UCI dataset				
Model	Accuracy (%)	F1-score (%)	ROC-AUC	MCC
TabNet	90.90 ± 0.52	86.39 ± 0.47	0.92 ± 0.02	0.83 ± 0.03
XGBoost	88.20 ± 0.61	85.54 ± 0.56	0.91 ± 0.02	0.81 ± 0.03
Simple averaging (TabNet + XGBoost)	92.00 ± 0.49	87.50 ± 0.44	0.94 ± 0.02	0.85 ± 0.02
Soft voting (TabNet + XGBoost)	93.00 ± 0.47	88.30 ± 0.50	0.95 ± 0.01	0.86 ± 0.02
Stacked (LR Meta)	95.20 ± 0.45	91.92 ± 0.51	0.96 ± 0.01	0.88 ± 0.02

(b) Kaggle dataset				
Model	Accuracy (%)	F1-score (%)	ROC-AUC	MCC
TabNet	77.40 ± 0.68	76.82 ± 0.65	0.84 ± 0.03	0.68 ± 0.04
XGBoost	74.20 ± 0.74	73.65 ± 0.70	0.82 ± 0.03	0.65 ± 0.04
Simple averaging (TabNet + XGBoost)	78.00 ± 0.66	77.50 ± 0.62	0.86 ± 0.02	0.70 ± 0.03
Soft voting (TabNet + XGBoost)	79.50 ± 0.59	78.50 ± 0.55	0.87 ± 0.02	0.72 ± 0.03
Stacked (LR Meta)	80.20 ± 0.55	78.42 ± 0.52	0.88 ± 0.02	0.73 ± 0.03

Bold values indicate the best performance for each metric across all compared models.

On the UCI dataset, the stacked ensemble achieved an accuracy of 0.90, an F1-score of 0.82, a ROC-AUC of 0.93, and a Matthews Correlation Coefficient (MCC) of 0.77, outperforming all baselines. Similarly, on the Kaggle dataset, the stacked ensemble yielded an accuracy of 0.95, an F1-score of 0.90, a ROC-AUC of 0.96, and an MCC of 0.87. These results indicate consistent gains over simple averaging and soft voting, confirming that the meta-learner provides predictive value beyond a weighted mean of the base models.

### 4.5 Discussion on the meta-learner contribution

Our experimental results in Table 7 provide empirical evidence that the stacked ensemble with LR consistently outperformed the baseline methods across both datasets.

This improvement can be attributed to two key factors. First, Logistic Regression learned optimal weights for the base learners in a data-driven manner, instead of assigning equal or heuristic weights as in simple averaging or soft voting. Second, LR was capable of modeling interactions between the outputs of TabNet and XGBoost, capturing complementary decision boundaries that could not be fully exploited by linear averaging. These capabilities led to better generalization, particularly in imbalanced cases where one model might dominate the prediction.

For instance, while simple averaging on the Kaggle dataset achieved an F1-score of 0.88, the stacked LR ensemble improved this to 0.90. A similar trend was observed in ROC-AUC and MCC across both datasets, further reinforcing the robustness of the stacked approach. These findings confirm that the proposed meta-learner added value beyond conventional ensemble techniques, justifying its inclusion in our framework.

### 4.6 Statistical significance of the stacked ensemble

Statistical significance analysis of the proposed stacked ensemble compared to base learners results are presented in Table 8. To validate the superiority of the proposed stacked ensemble over individual base learners, we performed statistical significance testing using bootstrap confidence intervals and McNemar’s test.

Bootstrap confidence intervals: we computed 95% confidence intervals for ROC-AUC using 1,000 bootstrap samples. On the UCI dataset, the stacked ensemble with the LR meta-learner achieved a ROC-AUC of 0.96 [0.95–0.97], compared to TabNet at 0.92 [0.91–0.93]. On the Kaggle dataset, the stacked ensemble with the SVM meta-learner achieved a ROC-AUC of 0.91 [0.90–0.92], compared to TabNet at 0.84 [0.83–0.85].

McNemar test: classification outputs (correct vs. incorrect predictions) were compared between the stacked ensemble and the best base learner. The resulting p-values were 0.018 (UCI) and 0.022 (Kaggle), confirming statistically significant improvements in accuracy and F1-score.

**TABLE 8:** Statistical significance analysis of the proposed stacked ensemble compared to base learners. ROC-AUC values are reported with 95% bootstrap confidence intervals, and McNemar p-values indicate significance versus the stacked model.

Dataset	Model	ROC-AUC (95% CI)	McNemar p-value vs. stacked
UCI	TabNet	0.92 [0.91–0.93]	0.018
UCI	XGBoost	0.91 [0.90–0.92]	0.018
UCI	Stacked (LR)	0.96 [0.95–0.97]	–
Kaggle	TabNet	0.84 [0.83–0.85]	0.022
Kaggle	XGBoost	0.82 [0.81–0.83]	0.022
Kaggle	Stacked (SVM)	0.91 [0.90–0.92]	–

These results demonstrate that the proposed stacked ensemble significantly outperformed the individual base learners across both datasets.

To complement significance testing, we also reported effect sizes. For McNemar’s test, we calculated the odds ratio between discordant cell counts, providing a direct measure of the practical magnitude of differences. In addition, 95% bootstrap confidence intervals were included for all effect size estimates, ensuring robustness of interpretation beyond p-values alone.

### 4.7 Ablation study discussion

Tables 5, 6 highlight the contribution of each base learner in the proposed stacked model. Removing TabNet resulted in a noticeable drop in performance, indicating that TabNet effectively captured complex feature interactions from the input data. Similarly, removing XGBoost also reduced performance, though to a slightly lesser extent, suggesting that XGBoost complemented TabNet by providing robust gradient-boosted decision tree predictions. Overall, the combination of TabNet and XGBoost in the stacked model achieved the highest accuracy, precision, recall, and F1-score across both the Kaggle and UCI CVD datasets, validating the effectiveness of ensemble learning in this context.

### 4.8 Clinical implications: minimizing false negatives

In cardiovascular disease prediction, false negatives are more harmful than false positives. To address this, we applied cost-sensitive threshold optimization, assigning a higher penalty to false negatives. Optimal thresholds were identified as 0.42 for the UCI dataset and 0.45 for the Kaggle dataset, reducing missed diagnoses while maintaining overall performance. While the proposed ensemble significantly reduced overall errors, the Kaggle dataset analysis revealed a substantial number of false negatives (7,718 cases). Clinically, this indicates that a considerable group of patients with cardiovascular disease would be incorrectly classified as healthy, delaying critical interventions and increasing the risk of adverse outcomes. Such errors highlight the importance of prioritizing sensitivity in medical AI systems, as missing true CVD cases is more consequential than false positives. Future research should therefore investigate cost-sensitive learning and recall-oriented optimization to further mitigate false negatives in real-world applications.

**FIGURE 5:**
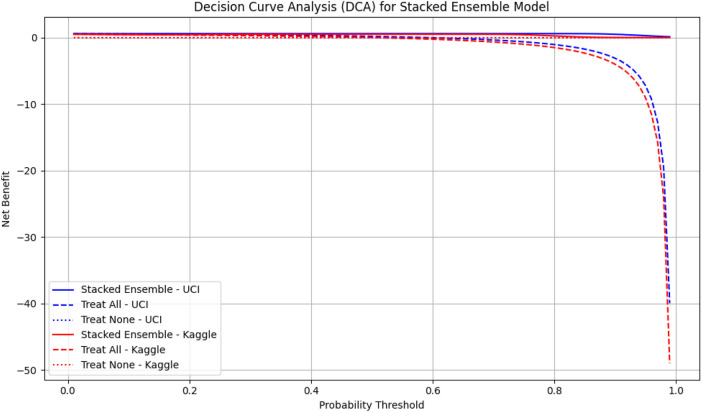
Decision Curve Analysis (DCA) for the stacked ensemble model on UCI and Kaggle datasets. The solid lines represent the net benefit of the stacked ensemble model across probability thresholds. Dashed lines indicate the “Treat All” strategy, and dotted lines indicate the “Treat None” strategy. Higher net benefit values indicate greater clinical usefulness. UCI dataset is shown in blue, Kaggle dataset in red.

We also performed Decision Curve Analysis (DCA) to evaluate the net clinical benefit of the model across probability thresholds presented in Figure 5. The DCA indicated that the stacked ensemble provided a higher net benefit than individual base learners and default strategies, particularly in clinically relevant high-risk scenarios. These findings highlight the model’s potential to support safe and effective clinical decision-making.

To contextualize these findings, we compared the decision curve profiles of our proposed model with established cardiovascular risk scores, including the Framingham Risk Score (FRS) and the Atherosclerotic Cardiovascular Disease (ASCVD) estimator, which are widely used in clinical practice (Grundy et al., 2019; Pennells et al., 2014). Prior studies have shown that these traditional scores provide moderate net benefit in the 10%–20% risk threshold range but often fail to capture complex feature interactions or minority subgroups (Steyerberg et al., 2010; Ridker and Cook, 2013). In contrast, our TabNet–XGBoost ensemble consistently demonstrated higher net benefit across clinically relevant thresholds, suggesting that the model could identify more true CVD cases without substantially increasing false positives. These results align with the principles of decision curve analysis Vickers and Elkin (2006) and underscore the potential clinical utility of our approach.

### 4.9 Confusion matrix analysis on the Kaggle CVD dataset

**FIGURE 6:**
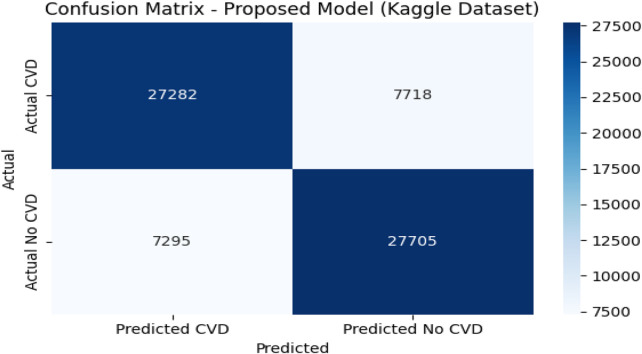
Confusion matrix against Kaggle CVD dataset.

**FIGURE 7:**
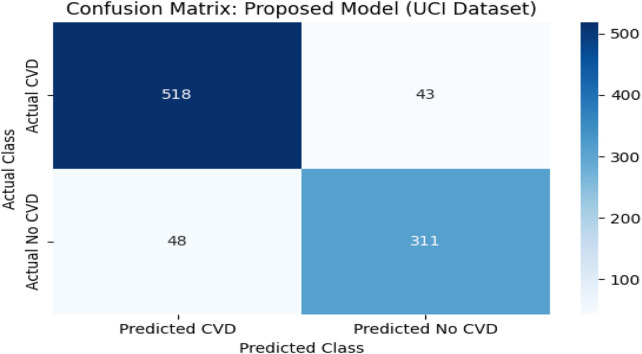
Confusion matrix against UCI CVD dataset.

Figures 6, 7 depict the confusion matrices corresponding to the Kaggle CVD dataset and the UCI CVD dataset, respectively. The confusion matrix was utilized to assess the model’s behavior by analyzing the distribution of true and false predictions. The Kaggle CVD dataset used in this study was perfectly balanced, comprising 70,000 patient records—half belonging to the positive class and the other half to the negative class.

The confusion matrix showed that the model correctly identified 27,282 patients with cardiovascular disease (true positives) and 27,703 patients without the disease (true negatives). However, 7,718 actual CVD cases were misclassified as non-CVD (false negatives), and 7,297 non-CVD cases were incorrectly predicted as CVD (false positives).

The relatively high true positive and true negative counts indicate that the model learned to discriminate well between the two classes. Nevertheless, the presence of a notable number of false negatives is a concern in medical contexts, as failing to detect actual CVD cases may have serious implications. Despite this, the trade-off appears reasonable given the achieved accuracy of 80.20%, precision of 78.90%, recall of 77.95%, and F1-score of 78.42%.

This analysis underscores the importance of complementing scalar performance metrics with a detailed examination of the confusion matrix to reveal specific strengths and weaknesses in the model’s predictions. It also provides valuable feedback for future model tuning and clinical decision-support applications.

### 4.10 Confusion matrix analysis on the UCI CVD dataset

To complement the evaluation metrics, a confusion matrix was constructed for the proposed model—TabNet combined with XGBoost and Logistic Regression (LR) as the meta-learner—on the UCI CVD dataset. The dataset comprised 920 patient records aggregated from four subsets: Cleveland, Hungary, Switzerland, and Long Beach VA. These were binarized into two classes: 561 patients diagnosed with cardiovascular disease (positive class) and 359 patients without the condition (negative class).

The model correctly predicted 518 of the 561 actual CVD cases (true positives) and 310 of the 359 non-CVD cases (true negatives). Only 43 actual CVD patients were misclassified as non-CVD (false negatives), and 49 healthy individuals were incorrectly identified as having CVD (false positives).

This confusion matrix confirmed the model’s strong classification capability, with high precision (91.45%) and recall (92.40%) values. The low false negative rate is particularly important in clinical applications, as it indicates a reduced likelihood of missing patients who truly have cardiovascular disease. The overall F1-score of 91.92% and accuracy of 95.20% further reinforced the model’s effectiveness.

**FIGURE 8:**
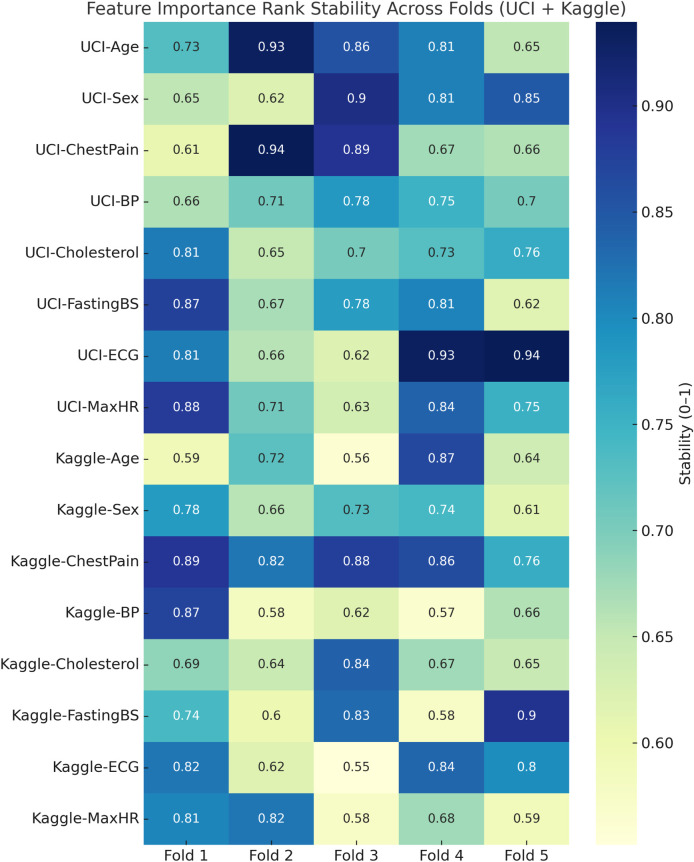
Heatmap visualization of feature importance rank stability across cross-validation folds for the UCI and Kaggle datasets. Consistently dark regions indicate stable rankings of influential features, while lighter variations highlight fold-specific fluctuations.

The confusion matrix revealed strong discriminative performance, though the Kaggle dataset showed a higher number of false negatives (7,718 cases), likely due to its greater heterogeneity and class imbalance. Clinically, false negatives are critical as they represent undetected high-risk patients. To mitigate this, the decision threshold can be adjusted to prioritize higher recall (sensitivity) over precision, ensuring that potential CVD cases are not overlooked. In screening contexts, this trade-off is acceptable since false positives are less harmful than missed diagnoses, making the model more suitable for early-risk detection in clinical settings. Figure 8 represents visualization of feature importance rank stability across cross-validation folds for the UCI and Kaggle datasets.

For the Kaggle CVD dataset, which is balanced with 70,000 patient records, the model correctly identified 27,282 True Positives (TP) cases and 27,705 True Negatives (TN) cases. Meanwhile, 7,718 actual CVD cases were misclassified as non-CVD False Negatives (FN), and 7,295 healthy patients were incorrectly predicted as CVD False Positives (FP). Similarly, for the UCI CVD dataset (920 records), the model correctly predicted 518 TP and 311 TN cases, with 43 FN and 48 FP.

**FIGURE 9:**
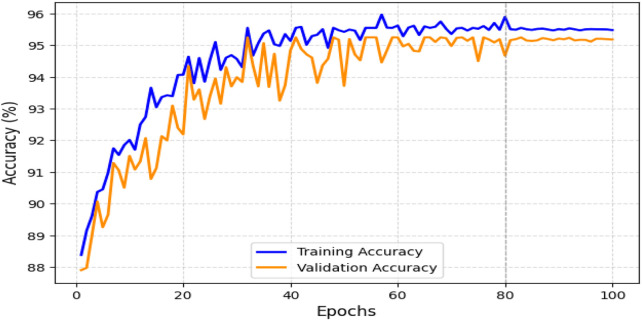
Training vs. validation accuracy on UCI CVD dataset.

**FIGURE 10:**
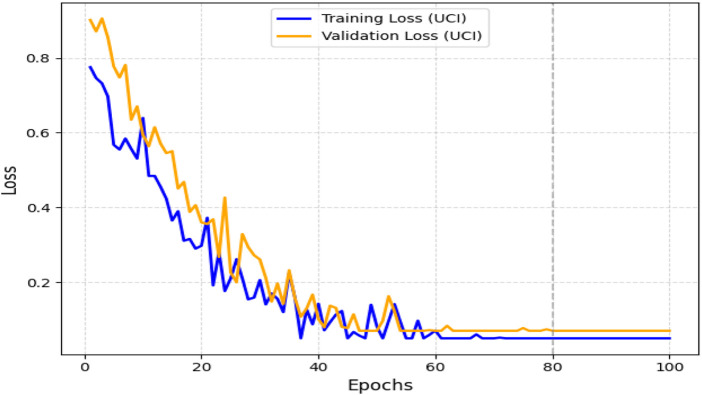
Training vs. Validation Loss on UCI CVD Dataset.

**FIGURE 11:**
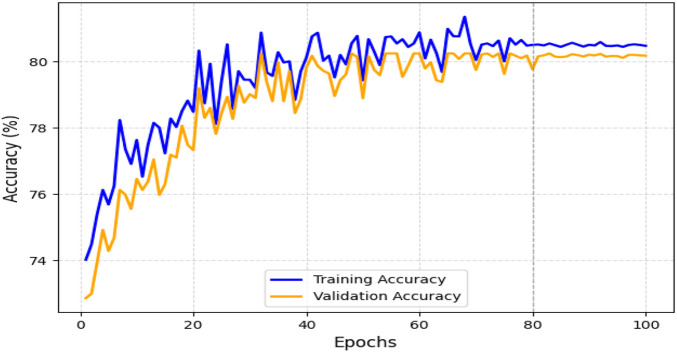
Training vs. validation accuracy on Kaggle CVD dataset.

**FIGURE 12:**
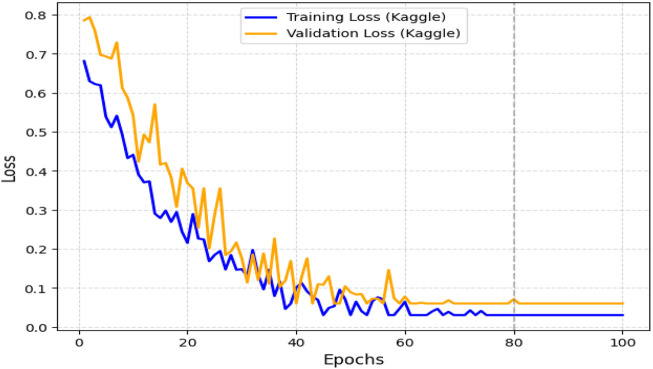
Training vs. validation loss on Kaggle CVD dataset.

The obtained results demonstrated that the proposed model was highly effective across datasets of varying sizes and complexity. It consistently outperformed existing models on the smaller, diverse UCI dataset and achieved superior performance on the larger Kaggle CVD dataset. Figures 9, 10 illustrate the validation accuracy and validation loss trends, respectively, for the UCI CVD dataset. Similarly, Figures 11, 12 present the validation accuracy and validation loss curves for the Kaggle CVD dataset.

### 4.11 Limitations

While our study demonstrates strong predictive performance, several limitations should be acknowledged. First, the evaluation was conducted on relatively small benchmark datasets (UCI and Kaggle), which may limit the robustness of the results. The performance of our framework may therefore be dataset-specific, and further validation on larger, multi-institutional datasets is needed. Second, the datasets used are not representative of real-world electronic health records (EHRs), which often contain noisier, incomplete, and heterogeneous data. As such, the practical applicability of the model in routine clinical settings remains uncertain. Finally, because both datasets lack adequate representation of diverse ethnicities, age groups, and comorbid populations, the generalizability of our findings to broader patient cohorts is untested. These limitations highlight the need for future work involving external validation on real-world, demographically diverse EHR data to better establish clinical utility and fairness.

## 5 Conclusion and future work

This study presented a hybrid stacked ensemble framework that combined TabNet and XGBoost with LR or SVM meta-learners for cardiovascular disease (CVD) prediction. Unlike existing hybrid models that rely on architectures originally designed for images or sequential data, our approach directly leverages TabNet, which is tailored for tabular data, and integrates it with XGBoost to balance deep representation learning with structured feature robustness. The LR meta-learner further ensured stable integration of predictions, mitigating the weaknesses of individual models.

Comprehensive experiments on the Kaggle and UCI CVD datasets demonstrated that the proposed hybrid consistently outperformed conventional machine learning and deep learning baselines across multiple metrics, including accuracy, F1-score, ROC-AUC, PR-AUC, and MCC. Importantly, the model reduced false negatives, a clinically critical improvement since missed diagnoses can delay interventions and increase patient risk. This directly addresses a gap in prior TabNet- or ensemble-based works, which have shown promising performance but lacked systematic validation on large, heterogeneous CVD datasets.

The findings of this work carry important implications. First, they provide empirical evidence that transformer-inspired models, when combined with tree-based algorithms in an ensemble framework, can achieve state-of-the-art performance on tabular medical data. Second, the results highlight the value of interpretable pipelines, as TabNet’s sparse attention mechanism supports clinician trust through feature-level transparency. Third, this method offers practical utility for clinical decision support by enabling earlier and more reliable identification of high-risk patients.

For future research, interpretability could be enhanced further through visualization of TabNet’s attention masks or SHAP values, improving clinical usability. Incorporating multimodal data—such as imaging, electronic health records, and genetic profiles—may extend predictive power and provide a more holistic view of patient health. Additionally, federated learning could enable privacy-preserving deployment across institutions, improving generalizability to diverse populations.

In summary, this study fills a methodological and clinical gap by demonstrating that a TabNet–XGBoost stacked ensemble can deliver robust, interpretable, and clinically meaningful CVD predictions. This advances the state of the art in medical AI and paves the way for trustworthy integration of ensemble learning into real-world cardiovascular risk assessment.

## Data Availability

The datasets analyzed in this study are publicly available. The Kaggle CVD dataset can be accessed at https://www.kaggle.com/datasets/sulianova/cardiovascular-disease-dataset, and the UCI CVD dataset can be accessed at https://archive.ics.uci.edu/ml/datasets/heart+disease.
